# Elastic deformation of the mandibular jaw revisited—a clinical comparison between digital and conventional impressions using a reference

**DOI:** 10.1007/s00784-021-03777-z

**Published:** 2021-01-13

**Authors:** Alexander Schmidt, Leona Klussmann, Maximiliane A. Schlenz, Bernd Wöstmann

**Affiliations:** Department of Prosthodontics, Dental Clinic, Justus Liebig University, Schlangenzahl 14, 35392 Giessen, Germany

**Keywords:** Clinical study, Digital dentistry, Dental impression technique, Full-arch impression, Dimensional measurement accuracy, Mandibular prosthesis

## Abstract

**Objectives:**

Due to the partly strongly differing results in the literature, the aim of the present study was to investigate a possible deformation of the mandible during mouth opening using an intraoral scanner (IOS) and a conventional impression for comparison with a reference aid.

**Materials and methods:**

Four steel spheres were reversibly luted in the mandibular (*n* = 50) with a metallic reference aid at maximum mouth opening (MMO). Two digital impressions (Trios3), at MMO and at slightly mouth opening SMO and a conventional impression (Impregum), were taken as the measuring accuracy of the reference structure was already known. Difference between MMO-SMO for digital impressions and deviations between digital and conventional (SMO) were calculated. Furthermore, the angle between the normal vectors of two constructed planes was measured. Statistical analysis was performed with SPSS25.

**Results:**

Deviations for linear distances ranged from −1 ± 3 μm up to 17 ± 78 μm (digital impressions, MMO-SMO), from 19 ± 16 μm up to 132 ± 90 μm (digital impressions, SMO), and from 28 ± 17 μm up to 60 ± 52 μm (conventional impressions, SMO). There were no significant differences for digital impressions (MMO-SMO), and there were significant differences between the conventional and digital impressions at SMO.

**Conclusions:**

Based on the results of the present study, no mandibular deformation could be detected during mouth opening with regard to the digital impressions. The results were rather within the measuring tolerance of the intraoral scanner.

**Clinical relevance:**

Based on the present study, no deformation of the mandibular during mouth opening could be observed at the level previously assumed. Therewith related, dental techniques related to a possible mandibular deformation therefore should be reconsidered.

## Introduction

The elastic deformation of the mandible during mouth opening has been a topic discussed for several decades. Moreover, this discussion continues to influence the education of dental students, and the daily work of dentists in routine dental practice and dental technicians in laboratories, especially when it comes to wide-span restorations. Therefore, for long-span fixed restorations in the mandible, split bridges are still fabricated to counteract the mandibular deformation during wide mouth opening [1–3].

However, most of the available data rely on studies from the last century [1, 4–11], which is the decisive aspect—show an extremely high variance with respect to the magnitude of the deformation observed. Goodkind and Herinlake found deviations ranging from 32 to 77 μm [7], whereas McDowell and Regli detected deviations up to 1400 μm [5].

Additionally, in many cases, the data are based on simple measuring devices and techniques. Although the approaches are relatively precise in their nature, they all are confronted with the problem of determining the exact situation in the mouth. Thus, most publications rely on the comparison of models made from conventional impressions (CVIs) recorded at different degrees of mouth opening [5, 8, 9, 11, 12].

Because these methods are not as precise as the modern digital techniques [6], the available data are contradictory. In addition, during impression taking and model fabrication, there could be possible errors in transfer accuracy already present in the impressions and models. Hence, for a precise evaluation of the transfer accuracy, an intraoral reference is necessary [13–15].

Due to the relatively different results in the literature, the aim of this study was to investigate the possible deformation of the mandible during mouth opening using a reference aid for comparison of between impressions with an intraoral scanner (IOS) and conventional technique.

The null hypothesis was that there is no statistical difference in the accuracy of the mandibular deformation measurement (trueness and precision) with respect to linear distances and possible torsions during mouth opening. A possible influence of female or male participants was investigated as covariate.

## Materials and methods

This clinical study included 50 volunteer participants (aged 18–36 years) with completely dentulous mandibles. Only those individuals with a minimal mouth opening capacity of 37 mm (incisal edge distance) were included, and the opening capacity ranged from 37 to 64 mm. The investigation was conducted in full accordance with the applicable ethical principles, including that of World Medical Association’s Declaration of Helsinki. The present study was approved by the local Ethics Committee of the Justus Liebig University (Giessen, Germany; Ref. no. 163/15). To ensure comparable test results [16], a single operator (L.K.) with experience in digital and conventional impression techniques performed all experiments.

**Fig. 1:**
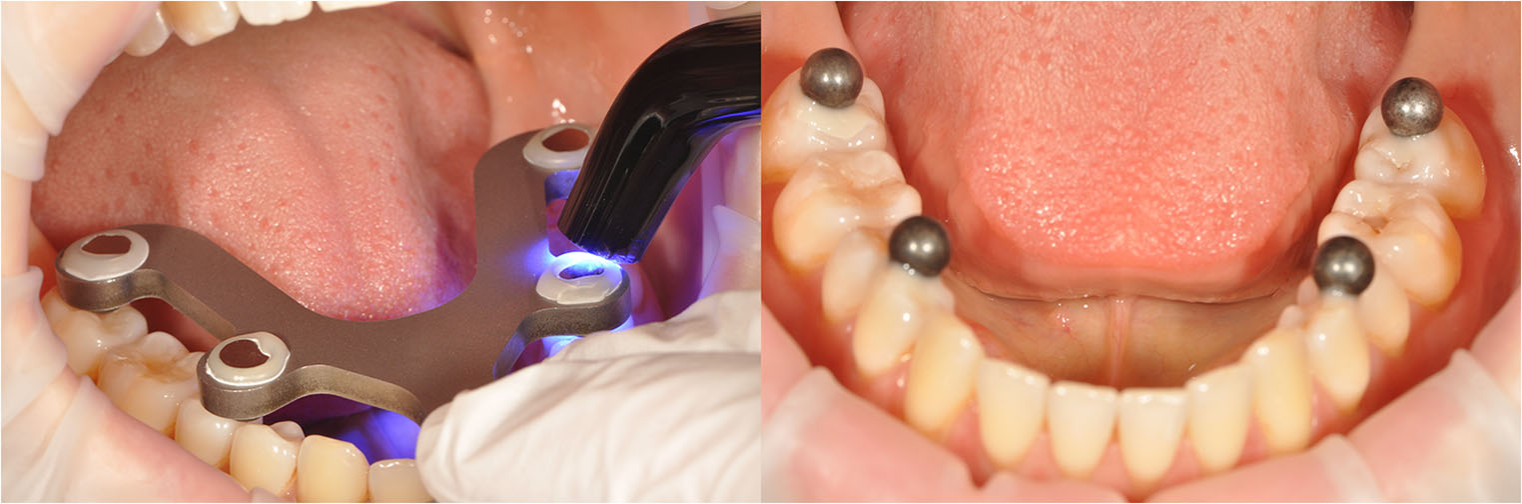
Fixation of the steel spheres using a metallic reference aid (at maximum mouth opening; left), and luting of steel spheres to the mandible (right)

For measuring purposes, four bearing steel spheres (diameter 5000 ± 5,63 μm; 1.3505 100Cr6 DIN5401 [17], ISO3290-01 [18]; TIS GmbH, Gauting, Germany) were reversibly luted to the mandibular teeth at MMO, using a flowable composite without prior etching (Grandio Flow, Voco, Cuxhaven, Germany, Fig. 1). A metallic reference aid (Bretthauer GmbH, Dillenburg, Germany) [14, 15] was used for exact positioning of the spheres, and a cheek retractor (Optragate, Ivoclar Vivadent, Schaan, Lichtenstein) was used to retract the cheeks and lips. The reference aid used was previously checked for fit in all participants. If the reference aid could not be positioned safely, the participant was excluded from the study.

This method is based on a previously described [14] and investigated technique [15]. In contrast to the previous study [14], high-precision spheres (roundness 5000 ± 5.63 μm ) were used [17, 18]. Thus, it was possible to enhance the precision of the placement of the luted spheres from 15 μm to less than 10 μm.

Subsequently, in every volunteer, two digital full-arch impressions were recorded using the Trios 3 Cart wired (software version 19.2.4, normal scan speed mode, manufactured 2016-03, 3Shape, Copenhagen, Denmark) at MMO and SMO. To obtain the best possible scan results under standardized conditions, the IOS systems were calibrated according to the manufacturer’s instructions. Scanning was started from the occlusal surfaces of the lower right quadrant to the lower left quadrant, followed by the oral surfaces and then the buccal surfaces [19]. The scan data were directly exported in a standard tessellation language (STL) dataset.

After removing the cheek retractor, a CVI was taken at SMO using a medium-body polyether impression material (Impregum Penta Soft Quick, batch no. 4811262, 3 M, Minneapolis, MN, USA) and a standard metal tray (Ehricke stainless steel, Orbis Dental, Münster, Germany). Before casting with type IV dental stone (Fujirock EP, batch no. 1810031, GC Corporation, Tokyo, Japan), the polyether impressions were stored for at least 2 h to ensure elastic recovery. The plaster models were stored under laboratory conditions (temperature, 23 ± 1 °C and humidity 50 ± 10%) for a minimum of 5 days.

To determine the dimensions of the reference aid, a coordinate measurement machine (CMM) (Thome Präzision GmbH, Messel, Germany) was used with the corresponding software (X4 V10 GA × 64, Metrologic Group, Meylan, France). For the reference dataset, the reference aid with the inserted spheres was measured 10 times, and the mean value for each sphere position was calculated. This digital reference model was stored as a dataset in the IGES format. Thereafter, each plaster model with respective spheres of the CVIs were measured 10 times with CMM, the mean value for each sphere was calculated and saved as digital datasets. The STL datasets of the digital impressions were imported into three-dimensional analysis software (GOM Inspect 2019, Gom GmbH, Braunschweig, Germany) for linear measurement between the centers of the spheres. The reference dataset of the reference aid was imported and saved as CAD-data in the GOM software. The imported STL dataset was saved as actual data. As the imported scan dataset only consists of a linked point cloud, four spheres were constructed using fitting elements (Gauß best fit, 3 Sigma) according to the scanned spheres. Subsequently, the deviations between the measured distances of the scan datasets and the reference aid data were calculated. The deviations in the MMO and SMO measurements were subtracted from the reference aid dimensions to obtain the relative deviation during mouth opening.

**Fig. 2:**
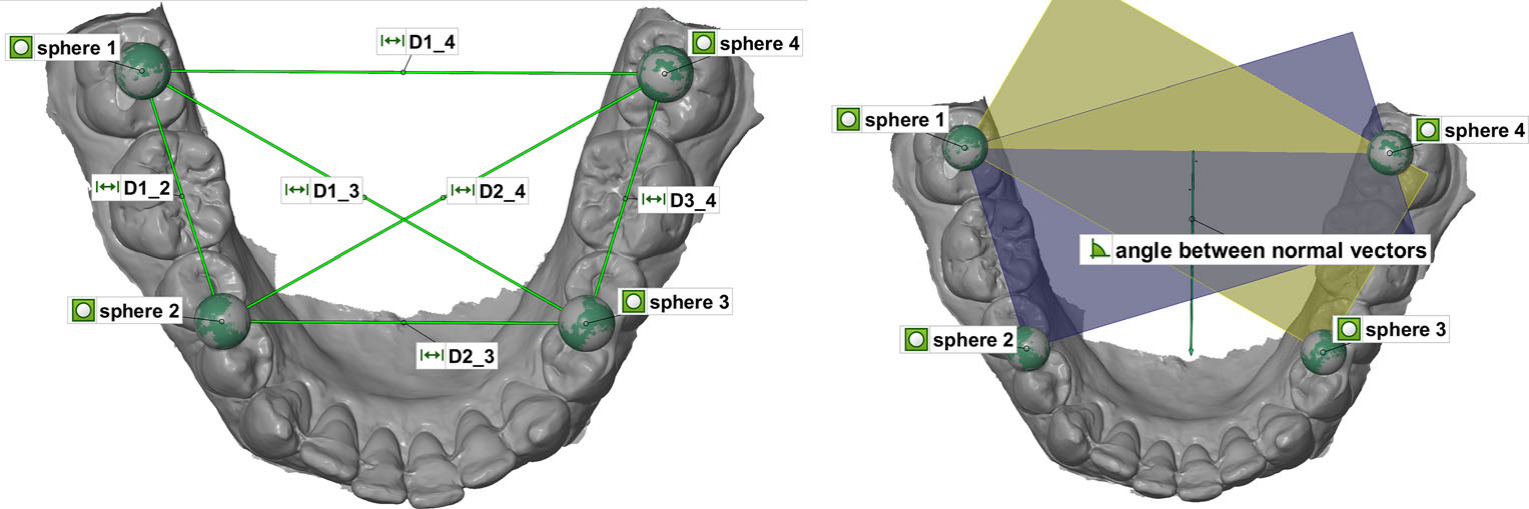
Representation of the measurement of linear distances (D1_2, D1_3, D1_4, D2_3, D2_4, and D3_4) between the centers of the four spheres (left) and measurement of the angle between the normal vectors (right)

Furthermore, a possible torsion during mouth opening was measured, and the angle in between the normal vectors of two constructed planes was measured (defined by spheres 1, 2, and 4 and 1, 3, and 4; Fig. 2).

**Fig. 3:**
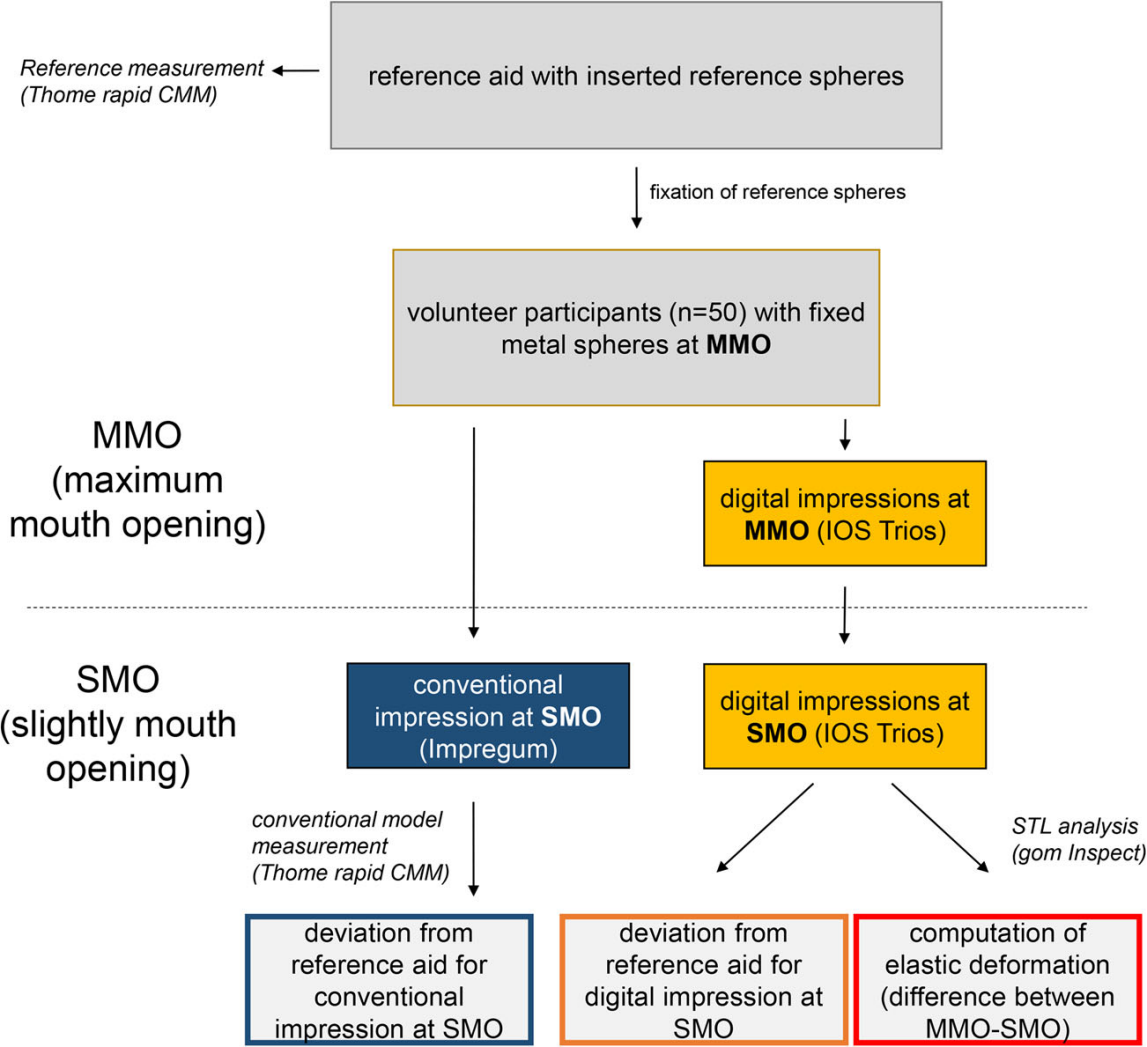
Overview of the entire measurement procedure

Summarized, a reference aid was luted at maximum mouth opening (MMO), and a digital full-arch impression was recorded with an IOS (Trios3) for MMO and slight mouth opening (SMO). The CVI (Impregum Penta) was taken for SMO, and a gypsum model was fabricated (Fujirock). Figure 3 shows an overview over the entire measurement procedure.

Statistical analysis was performed using SPSS version 25 (IBM Corporation, Armonk, NY, USA). The data were tested for normal distribution (Shapiro-Wilk and Kolmogorov-Smirnov Test, Lilliefors-corrected) and variance homogeneity (Levene test). According to ISO 5725-1, the mean values of the deviations between the impression results and the reference aid data described the trueness (mean) and the standard deviation (SD) described the precision [20]. Paired *t* tests were used to compare the differences in mean values between the different linear distances and angles under identical conditions (MMO, SMO, and CVI), as well as those between different conditions at identical distances and angles for dependent samples. Differences with *p* value < 0.05 were considered statistically significant. The Wilcoxon test was used when the test requirements were significantly violated due to outliers. Furthermore, an effect size *r* was computed for each difference. The interpretation was based on the suggestions of Kühnel and Krebs (*r* < 0.2, weak correlation; 0.2 < *r* > 0.5, medium correlation; and *r* > 0.5, strong correlation) [21]. To investigate a statistically significant difference between the genders (female, male) of the subjects, the mean equality of the differences between the two groups was tested using a *T* test on independent samples and the variance equality was investigated using the Levene test. For a better overview, the results are presented as box plot diagrams.

## Results

**Fig. 4:**
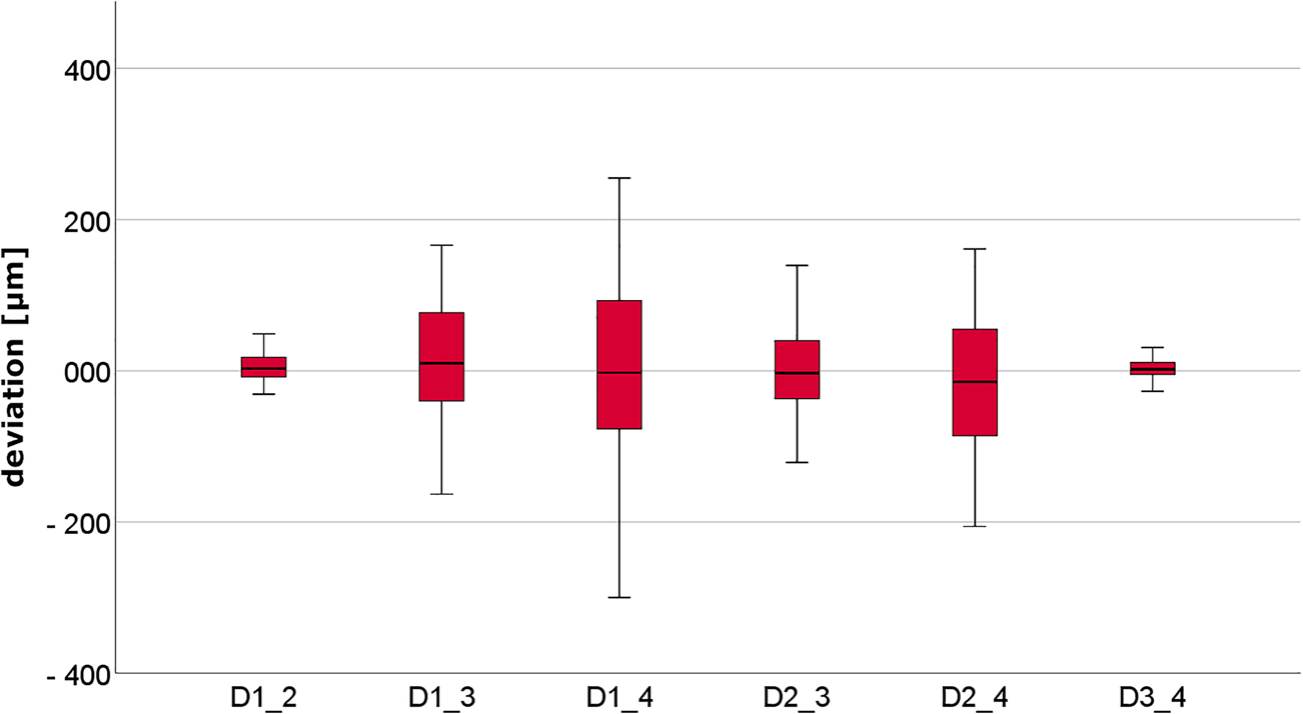
Box plot diagram of the deviations in the linear distances measured between the centers of the four spheres at maximal mouth opening (MMO) and slight mouth opening (SMO)

**Fig. 5:**
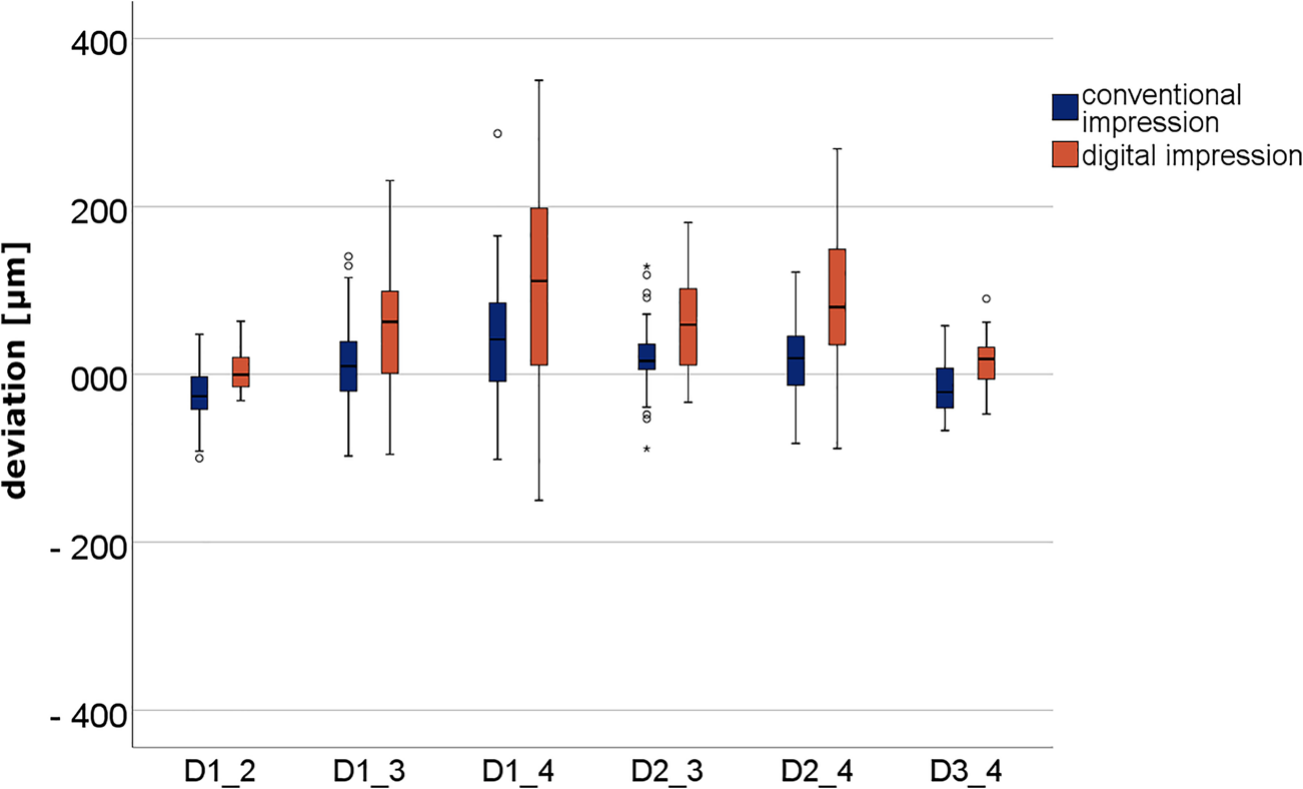
Box plot diagram of the deviations in the linear distances measured between the centers of the four spheres for conventional and digital impressions at slight mouth opening (SMO)

The digital impressions for MMO and SMO, and the CVIs for SMO resulted in 150 impressions from 50 volunteers. The calculated relative differences in the linear distances between the digital impressions at MMO and SMO are presented in Fig. 4 and Table 1. The results for the digital and conventional impressions at SMO are presented in Fig. 5 and Table 1.

**Table 1:** Deviations (mean ± standard deviation [μm]) in the linear distances (D1_2, D1_3, D1_4, D2_3, D2_4, and D3_4) and statistical analysis (significant differences *p* < 0.05 and strong correlation standard effect sizes *r* > 0.05) (presented in italics)

Linear distances	Method/impression technique		*p* value/*r* (effect size)	
		Mean (trueness) ±SD (precision) [mm]	MMO-SMO	SMO-CVI
D1_2	MMO-SMO	0.005 ± 0.017	0.913/0.016	*< 0.050*/0.380
	SMO	0.019 ± 0.016		
	CVI	0.032 ± 0.023		
D1_3	MMO-SMO	0.017 ± 0.078	0.513/0.094-	*< 0.001/> 0.05*
	SMO	0.076 ± 0.052		
	CVI	0.038 ± 0.032		
D1_4	MMO-SMO	− 0.008 ± 0.142	0.376/0.127	*< 0.001/> 0.05*
	SMO	0.132 ± 0.090		
	CVI	0.060 ± 0.052		
D2_3	MMO-SMO	− 0.001 ± 0.003	0.760/0.044	*< 0.001/> 0.05*
	SMO	0.065 ± 0.049		
	CVI	0.034 ± 0.030		
D2_4	MMO-SMO	− 0.020 ± 0.094	0.170/0.195	*< 0.001/> 0.05*
	SMO	0.095 ± 0.067		
	CVI	0.039 ± 0.029		
D3_4	MMO-SMO	0.002 ± 0.013	0.091/0.239	0.426/0.114
	SMO	0.025 ± 0.017		
	CVI	0.028 ± 0.017

For a better overview, deviations (mean ± SD [μm]) of the linear distances (D1_2, D1_3, D1_4, D2_3, D2_4, and D3_4) and statistical analysis (p values and effect size *r*) are presented in Table 1.

Except for D1_2 and D3_4 measurements, the CVI demonstrated the lowest deviation for all measurements.

For both MMO and SMO, the largest deviations were observed for distance D1_4 (intermolar distance).

The lowest deviations in the digital impressions for MMO and SMO were detected for both D1_2 and D3_4 (premolar-molar distances).

There were no statistically significant differences between the deviations at MMO and SMO in the digital impressions. However, partially significant differences were observed between the digital and conventional impressions at SMO.

**Fig. 6:**
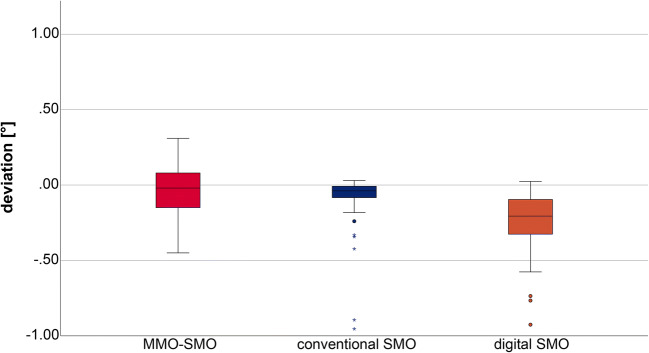
Box plot diagram of the deviations in the angle between the normal vectors of the two constructed planes (defined by spheres 1, 2, and 4 and 1, 3, and 4) showing difference between digital impressions at maximal mouth opening (MMO) and slight mouth opening (SMO), and between conventional and digital impressions at slight mouth opening

Furthermore, a possible torsion during mouth opening was measured. The angle measured between the normal vectors of the two constructed planes (defined by spheres 1, 2, and 4 and 1, 3, and 4) are presented in Fig. 6. For a better overview, deviations in the angle (mean ± SD [°]) and the statistical analysis (*p* values and effect size *r*) are presented in Table 2.

**Table 2:** Deviations (mean ± standard deviation [°]) in the angle between the normal vectors (defined by spheres 1, 3, and 4 and 1, 2, and 4) and the statistical analysis (significant differences *p* < 0.05 and strong correlation standard effect sizes *r* > 0.05) (presented in italics)

Angle between spheres	Impression technique		*p* value/*r* (effect size)	
		Mean ± SD [°]	MMO-SMO	SMO-CVI
1, 3, 4 and 1, 2, 4	MMO-SMO	-0.046 ± 0.164	0.057/0.268	*< 0.001/> 0.05*
	SMO	0.25 ± 0.21		
	CVI	0.11 ± 0.19		

The largest deviations in the angle at SMO were observed in the digital impressions. Moreover, significant differences were observed between the digital and conventional impressions at SMO.

There were no statistically significant differences between MMO and SMO for the digital impressions.

Statistical analysis of the covariate (female, male subjects) showed no significant differences between the groups.

The null hypothesis that there is no statistical difference in the mandibular deformation accuracy (trueness and precision, ISO 5725 [19]) in linear distances and possible torsions during mouth opening could not be rejected.

## Discussion

For this study, we optimized the method described by Kuhr et al. [14]. Besides the change in the measuring spheres (the previous study [14] used standard spheres as used in implantology) to high-precision spheres with extremely high roundness [17, 18], the temperature increase that occurs when changing from room to the oral situation was included in the creation of the reference data set. As the positioning plate is made of stainless steel, the material expands with an increase in temperature. The material-specific coefficient of thermal expansion was calculated, and the data obtained from the reference measurement with the CMM was corrected by the calculated expansion. In an in vivo study by Kameyama et al. [22], the oral temperature was measured in various participants during an investigation of several intraoral drainage methods. The highest measured temperature was 34 °C, which was confirmed in our preliminary tests. Hence, the oral temperature was fixed at this value and included in the calculation. The greatest thermal expansion for the longest distance (D1_4) was 8 μm, and this was within the measuring tolerance. Thus, we enhanced the accuracy with which the spheres were luted in place. This is reflected in the results of D1_2 and D3_4 (premolar to molar distance in the left and right quadrants, respectively).

The investigated IOS hardware and software components used in this clinical study are currently available in the market. Before application, the IOS system was calibrated according to the manufacturer’s specifications. For better comparison of the results with the current literature, an established methodology was used [14, 15], and the results were reported for trueness and precision in accordance with ISO 5725 as described previously in other studies [13, 14, 20].

Previous studies described the influence of the scan path on the accuracy of full-arch scans [19, 23, 24]. Therefore, a predetermined scanning protocol was used as recommended by Müller et al., who investigated different scan paths with the IOS Trios3 Pod [19].

With regard to the analysis of accuracy, trueness and precision was assessed according to ISO 5725-1 [20]. Although the method for describing trueness is generally agreed on, different approaches for the assessment of precision have been reported [25, 26]. We decided to use the ISO approach as a standardized method, which we consider helpful for a later comparison of our results with studies to come [13].

Comparing the results of the present study is difficult because, to our knowledge, no other study has investigated the mandibular deformation using an IOS. Moreover, only three studies have investigated full-arch impressions using a reference aid in patients [13–15]. Recent studies investigating mandibular deformation showed different results. With regard to CVIs, some investigations, such as those by Goodkind and Herinlake [7], showed results similar to that of the present study. They measured an anterior deviation of 32 μm and a posterior deviation of 77 μm. Fischman [27] also presented deviations of 71 ± 43 μm, comparable to our study. However, the majority of the studies showed a significantly higher deviation as compared to the results of this study. McDowell and Regli [5] observed MMO deviations of up to 1400 μm, while De Marco and Paine [28] observed deviations up to 1500 μm. Fischman [29] showed an average deviation of 860 ± 140 μm and Prasad et al. [30] from 390 μm up to 1120 μm. Shinkai et al. [12] performed impressions at three different mouth openings and could measure deviations of up to 360 μm. Nevertheless, these deviations were smaller in a follow-up study [31]. Deviations in the range of 140 up to 300 μm could also be shown in investigations of Custodio et al. [32] and Wolf et al. [33]. However, recent investigations were performed with fewer participants, and the measuring methods seemed less precise as compared to those used in the present study. The measuring problem can also be seen on the results of different studies on implants. Hobkirk and Schwab [34], Horiuchi et al. [1], Richter [10], Abdel-Latif et al. [35], and Al-Sukhun and Kelleway [36] fixed the measuring devices in the molar or premolar region on implants. Therefore, smaller deviations could be identified. However, in the case of a possible deformation, exactly the opposite were to be expected on implants, as the deformation that occurred can no longer be compensated by natural teeth.

In contrast, Horiuchi et al. [1] described deviations of two implants from each other at MMO, ranging between 7.8 and 24.6 μm. Moreover, the linear differences shown in the study by Abdel-Latif et al. [35] ranged between 1.4 and 41.3 μm, which was reflected in the results of Al-Sukhun and Kelleway [36] who identified deviations of 14.4–58.4 μm. According to these authors, the deviations could be attributed to a deformation of the mandible during MMO. Because the deviations are in the range of the natural periodontium’s own mobility, it is difficult to draw a comparison with the results of the present study.

In this study, no significant difference was detected between the deviations in the female and male participants, and this corroborates with the findings of Chen et al. [37] and Wolf et al. [33].

The deviations measured in the digital impressions for minimum mouth opening were not significantly different from those at MMO. This is clearly elicited in the differences between the results of MMO and SMO (Fig. 4). Here, it is obvious that the median values are almost all on the zero line, and thus no difference between MMO and SMO in terms of mandibular deformation is detectable. Although there is a high dispersion, this could be due to possible stitching or matching errors with increasing scan path length. This is particularly evident in the fact that the short distances of D1_2 and D3_4 hardly showed any deviations. These findings can be compared with the results of a previous study [15]. The longer distances, and also those that completely cross the quadrant (especially D1_4) showed the greatest scatter. These results are comparable with previous investigations [13–15]. In summary, the results of the digital impressions demonstrate that the IOS shows less deviation at short distances up to one quadrant compared to conventional impressions. For full-arch impressions, whether an intraoral scanner can be used for full-arch impressions depends on the definition or indication.

This is confirmed by the deviations in the CVIs. Furthermore, the scattering could be due to plaster expansion and polyether shrinkage, since scattering also occurred in the shorter distances (D1_2 and D3_4). The results were contrasting with the digital impressions of SMO. This confirms the results of a previous study [15] for short distances. Moreover, the spread can be attributed to difference among the patients, as shown by Hobkirk and Schwab [34], Richter [10], and Wolf et al. [33], who reported similar observations. It is noticeable that older investigations where the possible mandibular deformation was measured on models could show very high deviations. This can also be attributed to unavoidable deviations in the fabrication of impressions and plaster casts during extraoral measurements (e.g., plaster expansion and impression material shrinkage) or during intraoral measurements with older measuring instruments [4, 5, 28].

Thus, the present study does not confirm the previous results of mandibular deformation during mouth opening with deviations up to 1500 μm. In fact, the results were within the measuring tolerance of the digital and conventional impression methods currently used. Furthermore, it can be said from clinical experience that if deviations of the magnitude described in the literature were to occur, fixed or removable complete prosthetic restorations in the entire mandible would not be possible. As a limitation of the study, possible deformations of the mandible during mouth opening, but not during forward movements of the mandible, were examined.

## Conclusions

In summary, the significant change in the width of the mandible during MMO, which has been partially described in the literature, could not be confirmed in this clinical study. The possible deformations occurring in the mandible were within the possible measuring tolerance of the currently used digital and conventional impression methods.
